# Current status of neoadjuvant therapy for locally advanced rectal cancer in Wuhan Union Hospital Cancer Center

**DOI:** 10.1186/s13014-022-02081-8

**Published:** 2022-06-20

**Authors:** Meng-Lan Zhai, Fang-Yuan Zhang, Jin-Ru Yang, Sheng Zhang, Lei Zhao, Zhen-Yu Lin, Jing Wang, Dan-Dan Yu, Tao Zhang

**Affiliations:** Cancer Center, Tongji Medical College, Union Hospital, Huazhong University of Science and Technology, Wuhan, 430022 China

**Keywords:** Rectal cancer, Neoadjuvant therapy, Long course chemoradiotherapy, Short-course radiotherapy, Pathological complete response

## Abstract

**Background:**

To analyze and explore the evolution and short-term efficacy of neoadjuvant therapy for patients with mid and low LARC in Wuhan Union Hospital Cancer Center.

**Methods:**

Patients diagnosed with rectal cancer from January 2015 to December 2021 were collected. The treatment patterns, short-term efficacy and treatment-related adverse events (AEs) of mid and low LARC patients who received neoadjuvant therapy were analyzed. The Chi-square test was used to compare the differences between groups.

**Results:**

A total of 980 patients with mid and low LARC were enrolled, over time, the proportion of patients receiving neoadjuvant therapy gradually increased, and the treatment mode of direct surgery after diagnosis was gradually watered down. More than 80% of the patients implemented radiotherapy-based neoadjuvant therapy, and the proportion of patients receiving SCRT sequential systemic therapy gradually exceeded that of LCRT combined chemotherapy after 2020. Of all patients who completed radiotherapy and underwent surgery, 170 patients received long-course chemoradiotherapy (LCRT) combined with chemotherapy (Group C) and 98 patients received short-course radiotherapy (SCRT) combined with systemic therapy (chemotherapy with or without immunotherapy) (Group D). The pathological complete response (pCR) rate in Group D was significantly higher than that in Group C (38.8% vs. 19.4%, *P* = 0.001). The pCR rate in the SCRT plus immunotherapy group was better than that in the group without immunotherapy (49.2% vs. 21.6%, *P* = 0.007). 82.3% of the patients receiving immunotherapy were treated with SCRT sequential 2-cycle CapOX plus Camrelizumab treatment, and the pCR was as high as 52.9%. Immunotherapy did not increase the incidence of Grade 3–4 AEs.

**Conclusions:**

Neoadjuvant therapy based on radiotherapy is becoming used in patients with mid and low LARC. SCRT sequential systemic therapy is increasingly widely used in LARC patients in our center. Compared with the traditional LCRT or SCRT sequential chemotherapy, SCRT sequential chemotherapy plus immunotherapy has a remarkable pCR rate and manageable toxicity. Looking forward this new treatment mode will bring lasting survival benefits to patients.

**Supplementary Information:**

The online version contains supplementary material available at 10.1186/s13014-022-02081-8.

## Background

Colorectal cancer is the 2nd most common cancer and the 4th leading cause of cancer-related death in China [1]. It was reported that 72.2% of rectal cancer patients were diagnosed with locally advanced rectal cancer (LARC) at their first visit [2]. At present, neoadjuvant chemoradiotherapy (nCRT) combined with total mesorectal excision (TME) has become the standard treatment strategy for mid and low LARC [3]. Neoadjuvant chemoradiotherapy, as a key preoperative treatment for TME of mid and low LARC, not only helps to reduce the local recurrence rate, achieve tumor downstaging, and improve the R0 resection rate of surgery, but also enables some patients to achieve pathological complete response (pCR). In recent years, domestic and foreign researchers have conducted a large number of clinical studies on preoperative nCRT modalities, and the study data showed that about 8–48% of patients with mid and low LARC could achieve pCR after nCRT [4–8]. It can be seen that there are large individual differences in response to the efficacy of different neoadjuvant treatment modalities. Therefore, this study retrospectively analyzed the current status of treatment for mid and low LARC in Wuhan Union Hospital Cancer Center from January 2015 to December 2021, and the effects of different neoadjuvant treatment patterns on disease control and pathological response in the real world outside of clinical trials.

## Methods

### Patients

**Fig. 1:**
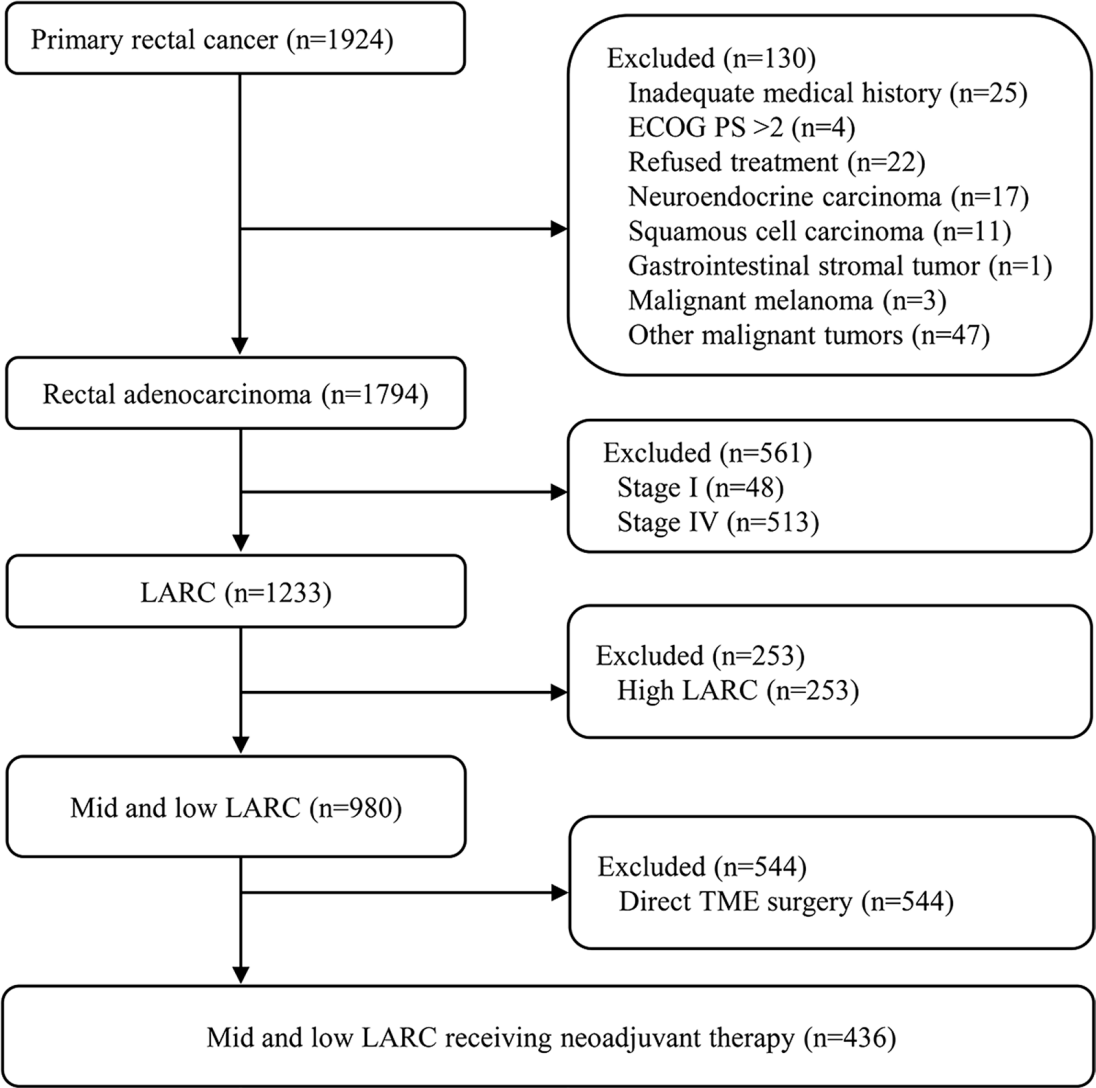
Flowchart of patients screening

Clinical information of 1924 patients with primary rectal cancer diagnosed in Wuhan Union Hospital Cancer Center from January 2015 to December 2021 was collected. Finally, a total of 980 patients with mid and low LARC were included in the analysis, of whom 436 patients received neoadjuvant therapy and the rest underwent direct surgery as initial treatment (Fig. 1). The clinical stage of rectal cancer at the time of initial diagnosis was confirmed according to the 8th edition of the International Union Against Cancer (UICC)/American Joint Committee on Cancer (AJCC) tumor-node-metastasis (TNM) staging system. Eligibility criteria included histopathological confirmed primary rectal adenocarcinoma, with the inferior border no more than 10 cm away from the anal verge; ultrasound endoscopy or magnetic resonance imaging (MRI) staged II (T3-4N0) or stage III (T1-4N1-2) with no evidence of distant metastasis; Eastern Cooperative Oncology Group (ECOG) performance status (PS) 0–1; No contraindications of radiotherapy and chemotherapy, molecular targeted therapy, immunotherapy and surgical treatment. Exclusion criteria included inadequate medical history information; ECOG PS > 1; Histopathological confirmed neuroendocrine carcinoma, squamous cell carcinoma and gastrointestinal stromal tumor; Complicated with other malignant tumors; patients with stage I and IV; with the lower border of the tumor more than 10 cm from the anal verge; TME surgery directly after diagnosis. All patients were given written informed consent for treatment and their medical data were used anonymously for medical research. This study was in accordance with the ethical standards of the institution and with the Declaration of Helsinki (as revised in 2013) and approved by the ethics committee of Union Hospital, Tongji Medical College, Huazhong University of Science and Technology.

### Treatment

Preoperative neoadjuvant radiotherapy includes LCRT and SCRT. LCRT was performed by three-dimensional conformal radiation therapy (3D-CRT) or volume intensity-modulated ARC therapy (VMAT) or intensity-modulated radiation therapy (IMRT). Mid and low LARC administered a total prescription irradiation dose of 45–50.4 Gy in 25–28 fractions, delivered in 1.8–2 Gy fractions daily on 5 consecutive days per week for 5–6 weeks. Synchronized 5-Fluorouracil (5-FU) or capecitabine-based chemotherapy for the duration of radiation, followed by TME surgery (no less than 3 weeks). SCRT adopted a total prescription irradiation dose of 25 Gy in 5 fractions for 5 consecutive days, without concurrent chemotherapy, followed by delayed TME surgery (more than 6 weeks). Neoadjuvant chemotherapy regimens mainly included capecitabine and oxaliplatin (CapOX) or standard oxaliplatin, leucovorin and 5-fluorouracil (FOLFOX4) and modified FOLFOX6 (mFOLFOX6). Short-course radiation therapy combined with systemic therapy refers to chemotherapy with or without Camrelizumab. In this study, the operation was performed by total mesorectal excision (TME).

The efficacy of neoadjuvant therapy was evaluated by a combination of preoperative ultrasound endoscopy, chest computed tomography, pelvic MRI and postoperative pathology.

### Grouping situation

Patients were divided into three periods based on the time of initial treatment: from 2015–2017, 2018–2019, 2020–2021 (from the beginning to the end of the year). According to the different treatment methods, patients were divided into direct surgical treatment and neoadjuvant therapy followed by TME surgery, the latter included neoadjuvant LCRT, neoadjuvant SCRT and neoadjuvant chemotherapy alone.

### Statistical analysis

The pCR rate was used to evaluate the efficacy of neoadjuvant therapy. pCR was defined as surgical specimens (including lymph nodes) without any residual cancer cells under the microscope (ypT0N0M0) [9]. The tumor downstaging was defined as the proportion of patients with ypT0-2N0M0 for those who underwent TME and assessed using the AJCC 8th staging system. Besides, the R0 resection rate and the incidence of treatment-related adverse events were also explored. AEs were classified according to Radiation Therapy Oncology Group/European Organization for Research and Treatment of Cancer (RTOG/EORTC) and National Cancer Institute Common Terminology Criteria for Adverse Events (CTCAE), version 5.0.

All statistical analyses were performed using SPSS version 25.0 software (IBM Corporation, Chicago, IL, USA). Continuous variables are represented by median (interquartile range), while categorical variables are expressed as frequencies or percentages. Categorical variables were tested by the Chi-square test or Fisher’s exact test. A two-sided *P* value < 0.05 was considered a statistically significant difference.

## Results

### Evolution of treatment models

**Fig. 2:**
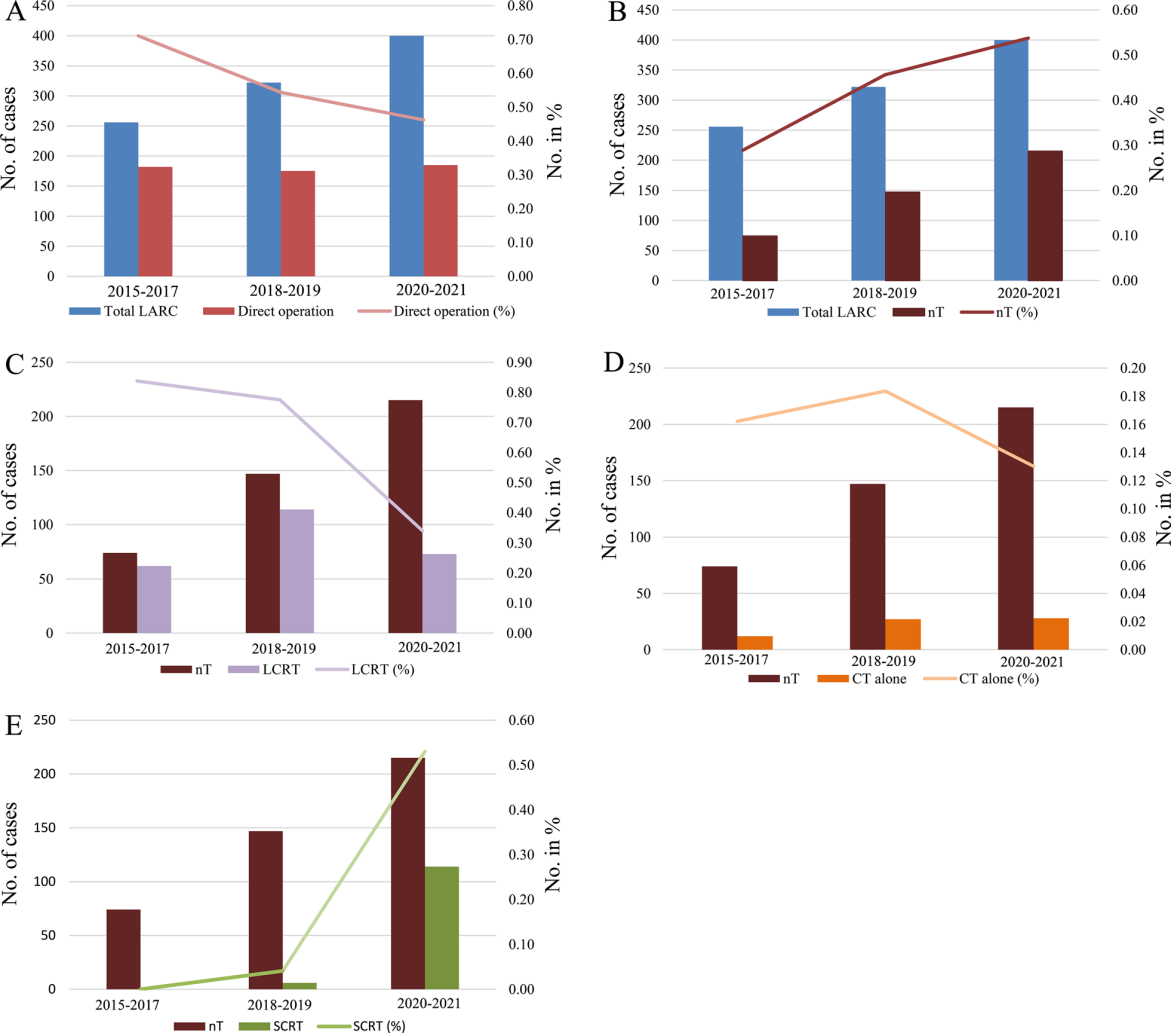
Trends in receipt of direct operation and preoperative neoadjuvant therapy for mid and low LARC between January 2015 and December 2021. **A**. The proportion of direct operation after diagnosis; **B**. The proportion of neoadjuvant therapy (nT); **C**. The proportion of neoadjuvant chemotherapy (CT) alone; **D**. The proportion of neoadjuvant short-course radiotherapy (SCRT); **E**. The proportion of neoadjuvant long-course chemoradiotherapy (LCRT);

From January 2015 to December 2021, a total of 980 patients with low and moderate locally advanced rectal cancer were admitted (Fig. 1). And 256, 322 and 400 patients were treated in 2015–2017, 2018–2019 and 2020–2021, respectively. The proportion of patients who underwent surgery directly after diagnosis was 71%, 54% and 46% (Fig. 2A), respectively, compared with 29%, 46% and 54% of patients receiving neoadjuvant therapy (Fig. 2B). The proportion of patients who received neoadjuvant LCRT during these three consecutive treatment periods was 84%, 78% and 34% (Fig. 2C), respectively, and the proportion of neoadjuvant chemotherapy alone was 16%, 18%, 13% (Fig. 2D). No neoadjuvant SCRT before 2019, SCRT was mainly concentrated in 2020–2021, accounting for 53% (Fig. 2E).

### Neoadjuvant therapy

**Fig. 3:**
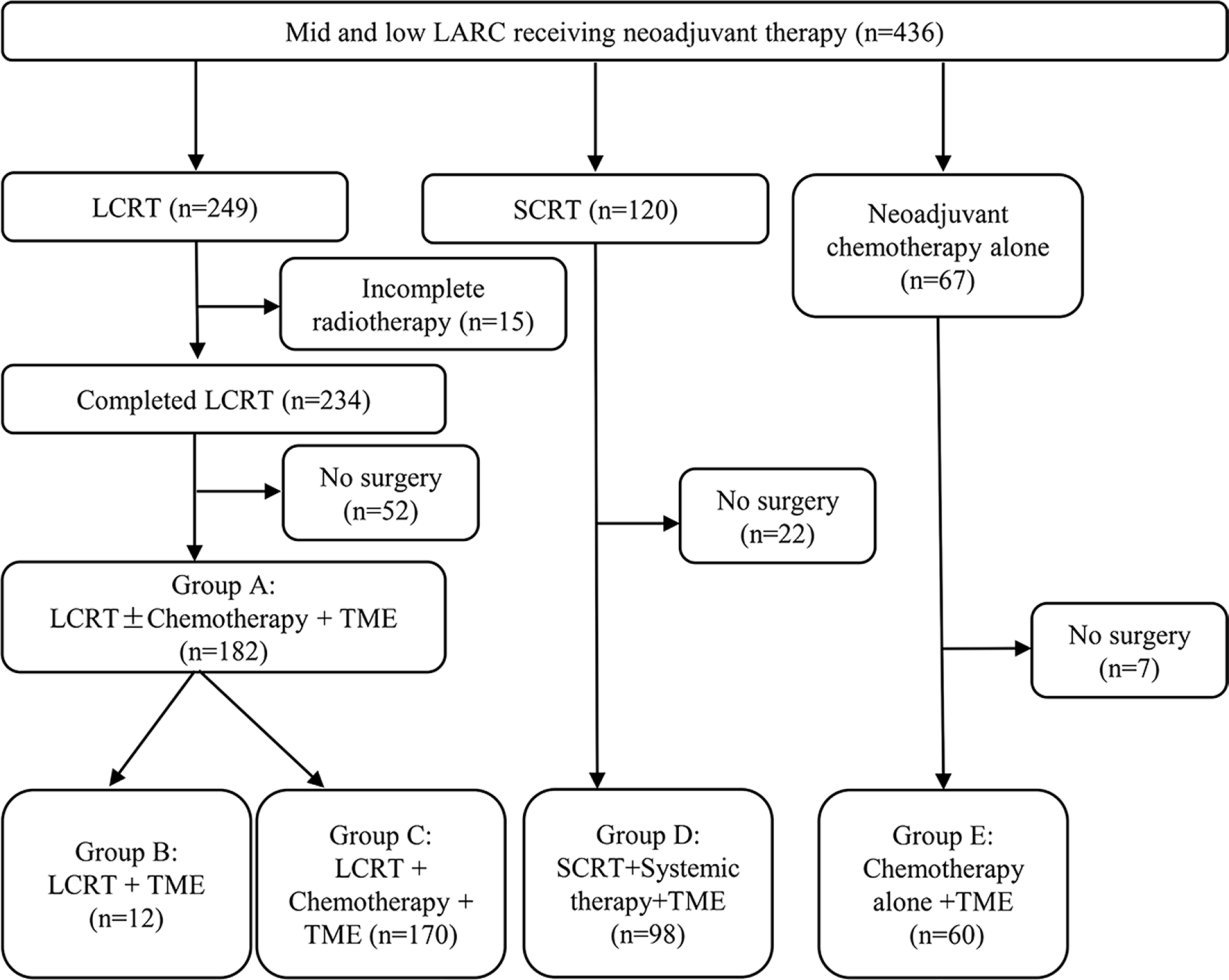
Neoadjuvant therapy mode for mid and low LARC

Of the 436 patients who received neoadjuvant therapy, 84.6% (369/436) of the patients were treated with standard neoadjuvant therapy based on radiotherapy (249 LCRT and 120 SCRT), while 15.4% (67/436) of the patients were treated with neoadjuvant chemotherapy alone (Fig. 3). Six percent (15/249) of patients who received LCRT failed to complete the full course of radiation therapy. In patients with SCRT, however, the completion rate of radiotherapy was 100%. For the above patients who completed LCRT, SCRT and simple chemotherapy, the operation implementation rate was 77.8%, 81.7% and 89.6%. From the records of patient’s clinical data, it was found that the reasons for the non-operation of mid and low LARC patients who completed neoadjuvant therapy could be summarized as follows: loss of follow-up, disease progression (distant metastasis) in the process of neoadjuvant therapy, treatment-related complication, refusal of surgery and some patients were still in the stage of neoadjuvant therapy (Additional file 1: Table S1). The basic characteristics of all patients who completed neoadjuvant therapy and underwent TME surgery were shown in Additional file 2: Table S2. Based on the neoadjuvant treatment modality, we divided the patients who underwent surgery into different groups: Group A, B, C, D, and E, as shown in Fig. 3. The number of patients in Group C and Group D accounted for the vast majority of the whole surgical population. The median time interval from the end of neoadjuvant radiotherapy to surgery was 9.4 weeks and 9.6 weeks in these two groups, respectively.

### Neoadjuvant therapy response rate

Considering that Group C and Group D were the main treatment modalities in this study, a subsequent analysis of these two groups of patients will be conducted. The pCR rates of Group C and Group D were 19.4% and 38.8% respectively, the difference was statistically significant (*P* < 0.05) (Table 1). The rate of N-stage decreased and R0 resection in Group C was slightly higher than those in Group D, the T-stage decreased rate was on the contrary, but the differences were not statistically significant (*P* > 0.05).

**Table 1:** The short-term efficacy of Group C and Group D

	Group C (n = 170)		Group D (n = 98)		*P* value
	No. of patients	%	No. of patients	%	
pCR	33	19.4	38	38.8	0.001
T-stage decreased	122	71.8	71	72.4	0.904
N-stage decreased	126	74.1	72	73.5	0.907
R0 resection	7	4.1	1	1.0	0.288

Abbreviations: pathological complete response

SCRT combined with systemic therapy modality (Group D) is divided into SCRT combined with chemotherapy modality (Group D1) and SCRT combined with chemotherapy plus immunotherapy modality (Group D2). Further analysis showed that the pCR rate of Group D2 was significantly higher than that of Group D1 (49.2% vs. 21.6%; *P* = 0.007) (Table 2). The rate of T-stage decreased rate in Group D2 was higher than those in Group D1 (80.3% vs. 59.5%; *P* = 0.025), however, there was no significant difference in the N-stage decreased rate. More than four-fifths (51/61) of the patients in Group D2 received neoadjuvant treatment modality of SCRT sequential 2-cycle CapOX plus Camrelizumab treatment. And the pCR rate of these patients was as high as 52.9% (27/51).

**Table 2:** The short-term efficacy of Group D1 and Group D2

	Group D1 (n = 37)		Group D2 (n = 61)		*P* value
	No. of patients	%	No. of patients	%	
pCR	8	21.6	30	49.2	0.007
T-stage decreased	22	59.5	49	80.3	0.025
N-stage decreased	26	70.3	46	75.4	0.576
R0 resection	0	100.0	1	1.6	-

Abbreviations: pathological complete response

### Neoadjuvant therapy safety

We have observed that the main side effects of neoadjuvant therapy are myelosuppression and radiation proctitis, so we will focus on this follow-up analysis. For patients who underwent surgery, the incidence of acute toxic side effects during neoadjuvant therapy was shown in Table 3. Granulocytopenia was the most common adverse event in grade 3–4. No grade 3 or more radiation proctitis occurred in all patients. The incidence of grade 1–2 radiation proctitis was 56.5% and 36.7% in Group C and Group D, respectively, with a significant difference (*P* = 0.002). According to medical records, no serious immune-related adverse events were observed in patients receiving immunotherapy. The probability of postoperative complications was 12.9% (22/170) and 19.4% (19/98) in Group C and Group D, respectively (*P* = 0.158) (Table 4). Postoperative complications were anastomotic stenosis (3 cases), leakage (1 case), wound infection (1 case), ileus (1 case) and urinary complications in Group D2.

**Table 3:** Acute adverse events (AEs) during neoadjuvant therapy in Group C and Group D

	Group C (n = 170)		Group D (n = 98)	
	Grade 1–2 (%)	Grade 3–4 (%)	Grade 1–2 (%)	Grade 3–4 (%)
Leukopenia	107 (62.9)	16 (9.4)	57 (58.2)	8 (8.2)
Neutropenia	76 (44.7)	19 (11.2)	42 (42.9)	13 (13.3)
Anemia	63 (37.1)	6 (3.5)	34 (34.7)	5 (5.1)
Thrombocytopenia	31 (18.2)	2 (1.2)	22 (22.4)	0 (0.0)
Radiation proctitis	96 (56.5)	0 (0.0)	36 (36.7)	0 (0.0)

**Table 4:** Postoperative complications in Group C and Group D

Postoperative complications	Group C (n = 44)	Group D (n = 38)
Leakage^a^	3	4
Anastomotic stenosis	4	3
Ileus	6	3
Wound dehiscence	3	2
Wound infection	2	1
Abnormal enterostomy^b^	2	4
Urinary complications	2	2
Overall postoperative complications	22	19

^a^Anastomotic leakage and other intestinal fistulas

^b^Parastomal hemias and Enterostomy stenosis

## Discussion

This study was a single-center retrospective study and the clinical data of 980 patients with mid and low LARC diagnosed and treated in the past 7 years were statistically analyzed. To some extent, it reflects the current situation of diagnosis and treatment of LARC patients in our cancer center. We all know that the MRC CR07 study and CAO/ARO/AIO-94 study established the position of neoadjuvant therapy in mid and low LARC treatment [4, 10], and the National Comprehensive Cancer Network (NCCN) and Chinese Society of Clinical Oncology (CSCO) guidelines recommended preoperative radiotherapy-based neoadjuvant therapy combined with TME as the standard treatment strategy for mid and low LARC. The results of this study showed that the implementation rate of preoperative neoadjuvant therapy gradually increased, while the proportion of surgery as the initial treatment showed a downward trend over time. This shows that the treatment of mid and low LARC by oncologists and surgeons in our center is becoming more and more standardized. In addition, more than 80% of patients were treated with standard neoadjuvant therapy based on radiotherapy. This is evident in the gradual increase in awareness and acceptance of neoadjuvant radiotherapy by physicians and patients.

All patients who underwent surgery were included in two dominant treatments: LCRT combined with neoadjuvant chemotherapy followed by TME and SCRT combined with systemic therapy followed by TME. It can be seen that the neoadjuvant therapy model of patients with mid and low LARC in this retrospective study was consistent with the standard treatment model recommended by the current major clinical practice guidelines [3, 11], which fully reflected that the level of diagnosis and treatment of LARC patients in our center was in line with international standards. While the vast majority of patients undergo neoadjuvant LCRT before 2020, the proportion of patients receiving LCRT declined sharply after 2020, conversely, there was a dramatic increase in patients receiving SCRT. This significant change in treatment mode may be influenced by a phase II clinical study of SCRT sequential 2-cycle CapOX plus immunotherapy conducted by our center. The pCR rate was 48% in this prospective study [8]. This kind of neoadjuvant therapy mode based on SCRT shows an amazing curative effect. In addition, short-course radiotherapy has the advantages of a large single fractionation dose, fewer fractionation times, short radiotherapy cycle, short hospitalization time and low cost, which greatly improves the cognition and acceptance of this treatment model.

In our study, we found that the pCR rate was significantly higher in the SCRT group (Group D) than in the LCRT group (Group C) (38.8% vs. 19.4%, *P* = 0.001). As we all know, the Polish II study was the first study of SCRT sequential chemotherapy versus LCRT [5], nevertheless, the results showed no significant difference in pCR rates between the two groups (16% vs. 12%, *P* = 0.17), but a lower rate of acute AEs with SCRT sequential chemotherapy compared with LCRT (75% vs. 83%, *P* = 0.006). Subsequently, in the RAPIDO trial [6], a total of 920 high-risk LARC patients were enrolled in SCRT combination total neoadjuvant therapy (TNT) versus standard LCRT, and preliminary data found that the pCR rate was significantly higher in SCRT sequential chemotherapy group than in LCRT group (27.7% vs. 13.8%, *P* < 0.001). The STELLAR trial [12], which used a non-TNT model, was equivalent to the RAPIDO Trial in terms of chemotherapy, and the final reports also suggested that SCRT combined with neoadjuvant chemotherapy had a higher pCR rate (16.6% vs. 11.8%) compared with LCRT. It can be seen that compared with LCRT, the PCR rate of the SCRT group (group C) is higher than that of other previous studies. Further analysis revealed that 62.2% of patients in the short course radiotherapy group received immunotherapy (Group D2) and 37.8% did not (Group D1). The pCR rate in Group D2 was also obviously better than that in Group D1 (49.2% vs. 21.6%, *P* = 0.007). 82.3% (51/61) of the patients receiving immunotherapy were treated with SCRT sequential 2-cycle CapOX plus Camrelizumab treatment, and the pCR was as high as 52.9%. In addition, immunotherapy did not increase the incidence of Grade 3–4 AEs. We speculate that immunotherapy may play a great role in this neoadjuvant therapy. The mechanisms involved in this high pCR rate will need to be further explored in the future. To this end, we look forward to the innovative phase III clinical trial (NCT04928807) of SCRT sequential chemotherapy plus immunotherapy conducted by our center will bring more benefits to patients with mid and low LARC.

Additionally, the dose of radiotherapy was found to be an important factor in the degree of tumor regression, and increasing the dose of concurrent radiotherapy helped improve the pCR rate with tolerable adverse effects [13, 14]. As we all know, the combination of radiotherapy and immune checkpoint inhibitors has synergistic antitumor effects [15, 16]. Preclinical studies suggest that single-dose > 5 Gy irradiation can effectively activate the immune response, combined with immune checkpoint inhibitors is expected to achieve a 1 + 1 > 2 effect [17]. The conventional 5 × 5 Gy mode was used for SCRT in this study. In the future, it may be possible to consider increasing the dose intensity of SCRT to explore the optimal splitting pattern in conjunction with chemotherapy plus immune checkpoint inhibitors.

There were still some limitations in this study. Firstly, this was a single-center retrospective study, and it was difficult to determine the effect of the sequence, regimen, and the number of cycles of neoadjuvant chemotherapy on outcomes for patients receiving neoadjuvant radiotherapy. Secondly, In the present study, the pCR rates of Group C and Group D1 were comparable, which may be limited by the small sample size. Hence, further expansion of the sample size is needed in the future to validate our conclusions. Despite these limitations, this retrospective study reproduces the current situation of diagnosis and treatment in the real world and has important clinical significance.

## Conclusions

This study was a real-world retrospective study that reflected the current status of neoadjuvant therapy for mid and low LARC in our cancer. Neoadjuvant therapy based on radiotherapy is becoming used in patients with mid and low LARC. SCRT sequential systemic therapy is growing widely used in LARC patients. Compared with the traditional LCRT or SCRT sequential chemotherapy, SCRT sequential chemotherapy combined with immunotherapy has a remarkable pCR rate and manageable toxicity. Looking forward this new treatment mode will bring lasting survival benefits to patients.

## Supplementary Information

**Additional file 1**. **Table S1**: The reasons why patients with LARC who completed neoadjuvant radiotherapy did not undergo surgery.

**Additional file 2**. **Table S2**: Patient characteristics.

## Data Availability

The datasets generated during and/or analyzed during the current study are available from the corresponding author on reasonable request.

## Abbreviations

LARC: Locally advanced rectal cancer
AEs: Adverse events
nT: Neoadjuvant therapy
LCRT: Long-course chemoradiotherapy
SCRT: Short-course radiotherapy
nCRT: Neoadjuvant chemoradiotherapy
TME: Total mesorectal excision
pCR: Pathological complete response
UICC: International Union against cancer
AJCC: American joint committee on cancer
TNM: Tumor-node-metastasis
MRI: Magnetic resonance imaging
ECOG: Eastern cooperative oncology group
PS: Performance status
3D-CRT: Three-dimensional conformal radiation therapy
VMAT: Volume intensity-modulated ARC therapy
IMRT: Intensity-modulated radiation therapy
5-FU: 5-Fluorouracil
CapOX: Capecitabine and oxaliplatin
FOLFOX4: Standard oxaliplatin, leucovorin and 5-fluorouracil
mFOLFOX6: Modified FOLFOX6
CT: Chemotherapy
RT: Radiotherapy
RTOG: Radiation therapy oncology group
EORTC: European organisation for research and treatment of cancer
CTCAE: Common terminology criteria for adverse events
NCCN: National comprehensive cancer network
CSCO: Chinese society of clinical oncology
