# Supplementary material for: Current status of neoadjuvant therapy for locally advanced rectal cancer in Wuhan Union Hospital Cancer Center
Source: Radiat Oncol. 2022 Jun 20;17:109. doi: 10.1186/s13014-022-02081-8 (PMC9208162; doi:10.1186/s13014-022-02081-8)
Supplement: Supplementary file 1 — Additional file 1. Table S1: The reasons why patients with LARC who completed neoadjuvant radiotherapy did not undergo surgery. [file 13014_2022_2081_MOESM1_ESM.docx]

**Table S1** The reasons why patients with LARC who completed neoadjuvant radiotherapy did not undergo surgery

|  | LCRT (n=52) | |  | SCRT (n=22) | |  | CT alone (n=7) | |
| --- | --- | --- | --- | --- | --- | --- | --- | --- |
|  | No. of Patients | % |  | No. of Patients | % |  | No. of Patients | % |
| Loss of follow-up | 8 | 15.4 |  | 1 | 4.5 |  | 3 | 42.9 |
| Distant metastasis | 6 | 11.5 |  | 3 | 13.6 |  | 1 | 14.3 |
| Treatment-related complication | 2 | 3.8 |  | 0 | 0.0 |  | 0 | 0.0 |
| Refusal of resection | 26 | 50.0 |  | 11 | 50.0 |  | 0 | 0.0 |
| Still in the neoadjuvant stage | 10 | 19.2 |  | 7 | 31.8 |  | 3 | 42.9 |

Abbreviations: LCRT: long course chemoradiotherapy; SCRT: short course radiotherapy; CT: Chemotherapy
