# Supplementary material for: Current status of neoadjuvant therapy for locally advanced rectal cancer in Wuhan Union Hospital Cancer Center
Source: Radiat Oncol. 2022 Jun 20;17:109. doi: 10.1186/s13014-022-02081-8 (PMC9208162; doi:10.1186/s13014-022-02081-8)
Supplement: Supplementary file 2 — Additional file 2. Table S2: Patient characteristics. [file 13014_2022_2081_MOESM2_ESM.docx]

**Table S2** Patient characteristics

| Characteristic |  | LCRT (Group A) | |  | SCRT (Group D) | |  | CT alone (Group E) | |
| --- | --- | --- | --- | --- | --- | --- | --- | --- | --- |
|  |  | No. of Patients | % |  | No. of Patients | % |  | No. of Patients | % |
| Age (years) |  |  |  |  |  |  |  |  |  |
| Median (range) |  | 54 (47-62) | |  | 55 (45-62) | |  | 57 (50.3-64.0) | |
| Gender |  |  |  |  |  |  |  |  |  |
| Male |  | 132 | 72.5 |  | 65 | 66.3 |  | 36 | 60.0 |
| Female |  | 50 | 27.5 |  | 33 | 33.7 |  | 24 | 40.0 |
| ECOG performance status |  |  |  |  |  |  |  |  |  |
| 0 |  | 34 | 18.7 |  | 15 | 15.3 |  | 9 | 15.0 |
| 1 |  | 148 | 81.3 |  | 83 | 84.7 |  | 51 | 85.0 |
| Compliations |  |  |  |  |  |  |  |  |  |
| 0 |  | 148 | 81.3 |  | 74 | 75.5 |  | 47 | 78.3 |
| ≥1 |  | 34 | 18.7 |  | 24 | 24.5 |  | 13 | 21.7 |
| T category |  |  |  |  |  |  |  |  |  |
| T2 |  | 4 | 2.2 |  | 2 | 2.0 |  | 3 | 5.0 |
| T3 |  | 109 | 59.9 |  | 72 | 73.5 |  | 41 | 68.3 |
| T4 |  | 69 | 37.9 |  | 24 | 24.5 |  | 16 | 26.7 |
| N category |  |  |  |  |  |  |  |  |  |
| N0 |  | 23 | 12.6 |  | 16 | 16.3 |  | 11 | 18.3 |
| N1 |  | 77 | 42.3 |  | 42 | 42.9 |  | 23 | 38.3 |
| N2 |  | 82 | 45.1 |  | 40 | 40.8 |  | 26 | 43.3 |
| Distance from tumor to anal verge (cm) |  |  |  |  |  |  |  |  |  |
| 0-5 |  | 110 | 60.4 |  | 50 | 51.0 |  | 29 | 48.3 |
| >5-10 |  | 72 | 39.6 |  | 48 | 49.0 |  | 31 | 51.7 |
| Mismatch repair status |  |  |  |  |  |  |  |  |  |
| pMMR |  | 121 | 66.5 |  | 84 | 85.7 |  | 53 | 88.3 |
| dMMR |  | 2 | 1.1 |  | 2 | 2.0 |  | 0 | 0.0 |
| Unkown |  | 59 | 32.4 |  | 12 | 12.2 |  | 7 | 11.7 |
| Circumferential resection margin (CRM) |  |  |  |  |  |  |  |  |  |
| Negative |  | 37 | 20.3 |  | 25 | 25.5 |  | 7 | 11.7 |
| Positive |  | 68 | 37.4 |  | 43 | 43.9 |  | 13 | 21.7 |
| Unkown |  | 77 | 42.3 |  | 30 | 30.6 |  | 40 | 66.7 |
| Extramural vascular invasion (EMVI) |  |  |  |  |  |  |  |  |  |
| Negative |  | 26 | 14.3 |  | 32 | 32.7 |  | 3 | 5.0 |
| Positive |  | 48 | 26.4 |  | 30 | 30.6 |  | 12 | 20.0 |
| Unkown |  | 108 | 59.3 |  | 36 | 36.7 |  | 45 | 75.0 |
| Neoadjuvant therapy model |  |  |  |  |  |  |  |  |  |
| RT alone |  | 12 | 6.6 |  | 0 | 0.0 |  | 0 | 0.0 |
| Induction CT+RT±consolidation CT |  | 66 | 36.3 |  | 9 | 9.2 |  | 0 | 0.0 |
| RT+consolidation CT |  | 104 | 57.1 |  | 89 | 90.8 |  | 0 | 0.0 |
| [Immunotherapy](javascript:;) |  | 0 | 0.0 |  | 61 | 62.2 |  | 0 | 0.0 |

Abbreviations: LCRT: long course chemoradiotherapy; SCRT: short course radiotherapy; CT: Chemotherapy；RT: Radiotherapy;

CRM: Circumferential resection margin; EMVI: Extramural vascular invasion
